# Emerging Verbal Functions in Early Infancy: Lessons from Observational and Computational Approaches on Typical Development and Neurodevelopmental Disorders

**DOI:** 10.1007/s41252-022-00300-7

**Published:** 2022-10-25

**Authors:** Peter B. Marschik, Claudius A. A. Widmann, Sigrun Lang, Tomas Kulvicius, Sofie Boterberg, Karin Nielsen-Saines, Sven Bölte, Gianluca Esposito, Anders Nordahl-Hansen, Herbert Roeyers, Florentin Wörgötter, Christa Einspieler, Luise Poustka, Dajie Zhang

**Affiliations:** 1 Child and Adolescent Psychiatry and Psychotherapy, Göttingen, Germany and Leibniz ScienceCampus Primate Cognition, University Medical Center Göttingen, Göttingen, Germany; 2 iDN - Interdisciplinary Developmental Neuroscience, Division of Phoniatrics, Medical University of Graz, Graz, Austria; 3 Research in Developmental Disorders Lab, Department of Experimental Clinical and Health Psychology, Faculty of Psychology and Educational Sciences, Ghent University, Ghent, Belgium; 4 Department of Pediatrics, David Geffen UCLA School of Medicine, Los Angeles, CA, USA; 5 Center of Neurodevelopmental Disorders (KIND), Centre for Psychiatry Research, Department of Women’s and Children’s Health, Child and Adolescent Psychiatry, Region Stockholm, Karolinska Institutet & Stockholm Health Care Services, Stockholm, Sweden; 6 Curtin Autism Research Group, Curtin School of Allied Health, Curtin University, Perth, WA, Austria; 7 Affiliative Behavior and Physiology Lab, Department of Psychology and Cognitive Science, University of Trento, Trento, Italy; 8 Department of Education, ICT and Learning, Østfold University College, Halden, Norway; 9 Third Institute of Physics-Biophysics, Georg-August University Göttingen, Göttingen, Germany; 10 Bernstein Center for Computational Neuroscience Göttingen, Göttingen, Germany

**Keywords:** Autism, Developmental disorder, Infant, Speech-language, Vocalisation

## Abstract

**Objectives:**

Research on typically developing (TD) children and those with neurodevelopmental disorders and genetic syndromes was targeted. Specifically, studies on autism spectrum disorder, Down syndrome, Rett syndrome, fragile X syndrome, cerebral palsy, Angelman syndrome, tuberous sclerosis complex, Williams-Beuren syndrome, Cri-du-chat syndrome, Prader-Willi syndrome, and West syndrome were searched. The objectives are to review observational and computational studies on the emergence of (pre-)babbling vocalisations and outline findings on acoustic characteristics of early verbal functions.

**Methods:**

A comprehensive review of the literature was performed including observational and computational studies focusing on spontaneous infant vocalisations at the pre-babbling age of TD children, individuals with genetic or neurodevelopmental disorders.

**Results:**

While there is substantial knowledge about early vocal development in TD infants, the pre-babbling phase in infants with neurodevelopmental and genetic syndromes is scarcely scrutinised. Related approaches, paradigms, and definitions vary substantially and insights into the onset and characteristics of early verbal functions in most above-mentioned disorders are missing. Most studies focused on acoustic low-level descriptors (e.g. fundamental frequency) which bore limited clinical relevance. This calls for computational approaches to analyse features of infant typical and atypical verbal development.

**Conclusions:**

Pre-babbling vocalisations as precursor for future speech-language functions may reveal valuable signs for identifying infants at risk for atypical development. Observational studies should be complemented by computational approaches to enable in-depth understanding of the developing speech-language functions. By disentangling features of typical and atypical early verbal development, computational approaches may support clinical screening and evaluation.

Myriad studies have furthered our understanding of the ontogeny of human behaviour and early neurofunctions that underlie our later capacities and skills. Early human behaviours are complex, dynamic, and diverse. Given commonalities in emerging neurofunctions along development, there are undeniable individual distinctions. One of the most fascinating questions in development is if, which, and how individual oscillations lead to long-term favourable or adverse outcomes? Following a neurodevelopmentalist perspective of development, acknowledging early functions as precursors and prerequisites for later ones, we presume that early deviations or impairments precede suboptimal traits or adverse outcomes, even if the core symptomatology of certain disorders may appear later in development (as for example in the case of autism spectrum disorder, ASD; e.g. Estes et al., 2019). This assumption, also known as deep constructivist notion (neuroconstructivism; e.g. Johnson, 2000; Johnson et al., 2021; Karmiloff-Smith, 1998; Mareschal, 2011; Westermann et al., 2011), is tightly linked to attempts at detecting and defining early functional markers of neurodiversity or atypicality, i.e. predictors of developmental trajectories (D’Souza & Karmiloff-Smith, 2017; Jones et al., 2014; Karmiloff-Smith, 1998, 2009; Marschik et al., 2014b, 2017; Micai et al., 2020). Concerns on whether behaviours reflect potential developmental atypicality or delay or mere diversity in typical development often result from recognising inter-individual discrepancies among peers caused by slowed or divergent functional acquisition within and across developmental domains, which might indicate stagnation or regression of intra-individual development. Notably, although the very early periods of speech-language development are not yet fully understood, atypicalities in the verbal domain are often one of the first perceived signs of neurodiversity during the first year of life.

Taking a closer look at the developing speech-language and communicative system, there is broad consensus regarding the essential role of prelinguistic vocalisations during early infancy for successful development of subsequent verbal functions (e.g. Karmiloff & Karmiloff-Smith, 2002; Locke, 1995; Oller, 2000; Vihman et al., 1985). Verbal development, meaning speech-language and communicative functions, follows a developmental trajectory of increasing complexity, accuracy and stability, thus building the complex human verbal capacity (e.g. Buder et al., 2013; Locke, 1995; Nathani et al., 2006; Oller, 1978, 2000; Papoušek, 1994; Stark, 1980). About four decades ago, stage-models were proposed describing developmental pathways from an infant’s first cry to becoming a competent communicator (c.f. Karmiloff & Karmiloff-Smith, 2002; Koopmans-van Beinum & van der Stelt, 1986; Oller, 1978; Papoušek, 1994; Roug et al., 1989; Stark, 1980). While there are differences in exact definitions and labels for categorically distinct vocalisation types, reported age of onset and stages/phases, and the mastering of certain milestones, researchers have offered similar models which describe evolving verbal functions. In the initial developmental phase, most vocalisations are faint and brief quasi-vowels. This first phase is often referred to as phonation stage or uninterrupted phonation stage (Fig. 1; Koopmans-van Beinum & van der Stelt, 1986; Oller, 2000). Thereafter, emerging at 1 to 2 months of age, vocalisations with articulatory movements of the tongue during phonation are uttered, a stage which was labelled “cooing” or “gooing” phase (Oller, 1978, 2000). Approximately 2 months later, an expansion of vocal and articulatory capacities can be observed. Vocalisation types at this expansion or vocal play stage, are vowel-/consonant-like sounds, squeals, and marginal syllables. These utterances are not yet produced with the articulatory accuracy and timing of adult-speech (Fig. 1; Nathani et al., 2006; Oller, 2000; Stark, 1980). The final stage of prelinguistic development, commonly referred to as canonical babbling stage, marks an infant’s start to produce speech-like syllables, usually starting between 5 and 10 months of age (Oller, 2000). Vocalisations are single or multiple consonant–vowel-combinations with rapid formant transitions between the consonantal and vocalic part. In some stage models, reduplicated and variegated babbling have been proposed as subsequent stages (Oller, 1978; Roug et al., 1989; Stark, 1980). In summary, specific vocalisation types occur in a cascading fashion and become increasingly speech-like towards the end of the first year of life, when the first (proto-)words are uttered. Besides this shift to language-specific phonetic forms, vocalisation types and developmental stages during the first year of life have been considered as universal (cf. Buder et al., 2013 who provide an acoustic phonetic catalogue of pre-speech vocalisations).

The classical approach to assess whether the above-mentioned early speech-language milestones are met, follows a perceptual segmentation-annotation-classification procedure of infant utterances. In such studies (which are observational), vocalisation-entities are commonly defined through the breath group criterion (i.e. vocalisation(s) uttered in the exhalation/expiration phase of one breathing cycle; Lynch et al., 1995a; Nathani & Oller, 2001) and segmented accordingly. Other approaches segmenting infant speech have differentiated vocalisations through a pause criterion (e.g. pauses longer 300 ms subdivide vocalisation clusters; Oller et al., 2010). In either way, the segmentation step is usually followed by an annotation process, in which trained listeners assign vocalisations to the predefined vocalisation classes (e.g. Koopmans-van Beinum & van der Stelt, 1986; Lang et al., 2021; Lynch et al., 1995a; Nathani et al., 2006; Oller, 1978, 2000; Roug et al., 1989; Stark, 1980). Recently, a citizen science study externally validated the expert classification of babbling vocalisations and the onset of canonical babbling (Cychosz et al., 2021). Together with findings on auditory Gestalt perception of experts and naïve listeners differentiating early verbal functions of infants with neurodevelopmental disorders (NDDs), this points to the existence of an intrinsic human Gestalt of different vocal categories or typical vs. atypical pre-linguistic vocalisations (Marschik et al., 2012a). Human auditory Gestalt perception, or the adult capacity of intuitively recognising different vocal categories, becomes more robust when evaluating “higher order verbal functions” of infants. Explicitly, babbling vocalisations, being more salient in form, are easier to be categorised by listeners as compared to pre-babbling vocalisations uttered in the first 5 months of life (e.g. Marschik et al., 2012a; Pokorny et al., 2018).

In the first 5 months of life, before the canonical babbling stage, the various stage-models concordantly include descriptions of a developmental pathway from simple phonation to an expansion phase (Fig. 1, e.g. Kent, 2022; Nathani et al., 2006; Oller, 2000). Oller and colleagues introduced a classification scheme of three types of infant vocalisations: cry, laughter and protophones; the latter are defined as precursors to speech and subdivided into vocants, squeals and growls (Jhang & Oller, 2017; Oller et al., 2013). Interestingly, evidence showed spontaneously produced protophones to outnumber cries and laughter from early on (Jhang & Oller, 2017; Oller et al., 2019). The importance of protophones lies, in contrast to cry and laughter, in their functional flexibility. They can be used in variable contexts and may fulfil different communicative functions (Jhang & Oller, 2017; Oller et al., 2013). Besides flexibility in functioning, the ontogeny of vocalisations has been discussed in terms of physiological constraints. Physiological adaptation of peripheral anatomical structures, such as the larynx descent or vocal-tract shape (e.g. Fitch, 2010; Lieberman et al., 2001) as well as neurophysiological changes governing the functional output, shape the development and the increasing complexity of vocalisations (see Fig. 1; e.g. Kent, 2021, 2022; Oller, 2000; Zhang & Ghazanfar, 2020).

In infants with various developmental disorders (DDs), an increasing number of studies has investigated the prelinguistic development aiming to detect early atypical findings and potential associations with later speech-language development (for reviews see for example Lang et al., 2019; Roche et al., 2018; Yankowitz et al., 2019). Canonical babbling, for example, was reported to be delayed or deviant in infants with hearing impairment (HI; Eilers & Oller, 1994; Koopmans-van Beinum et al., 2001; Moeller et al., 2007; Nathani Iyer & Oller, 2008; Shehata-Dieler et al., 2013; von Hapsburg & Davis, 2006), Down syndrome (DS; Lohmander et al., 2017; Lynch et al., 1995b), cerebral palsy (CP; Levin, 1999; Nyman & Lohmander, 2018), Williams-Beuren syndrome (WBS; Masataka, 2001), Cri-du-chat syndrome (CDS; Sohner & Mitchell, 1991), tuberous sclerosis complex (TSC; Gipson et al., 2021), autism spectrum disorder (ASD; Patten et al., 2014; Paul et al., 2011; Yankowitz et al., 2022), Rett syndrome (RTT; Einspieler et al., 2014; Marschik et al., 2012b, 2013), and fragile X syndrome (FXS; Belardi et al., 2017; Marschik et al., 2014a). Findings were however inconsistent and may depend on measures applied. For example, some infants with late detected developmental disorders (LDDDs such as ASD, RTT, FXS) exhibited a delayed onset of canonical babbling whereas others have reached this milestone at an adequate age, i.e. between 5 and 10 months (Bartl-Pokorny et al., 2022; Lang et al., 2019; Marschik et al., 2013; Yankowitz et al., 2019, 2022).

As findings regarding achievement of developmental milestones in infants with DDs were inconclusive, recent research increasingly aimed at gaining in-depth knowledge about early vocal patterns through the extraction and characterisation of acoustic features of emerging verbal functions. For example, in cry but also in spontaneous infant vocal patterns acoustic features like fundamental frequency (lowest frequency of a periodic waveform, usually denoted as *F*_0_) or duration of vocalisations have been documented (Borysiak et al., 2017; Buder et al., 2013; Hamrick et al., 2019; Kent & Murray, 1982; Wermke & Robb, 2010). More complex models on analysing acoustic properties of infant vocalisations include machine learning approaches applied on a set of parameters or features on signal level (Pokorny et al., 2020; Schuller & Batliner, 2013). There are established parameter sets for analysing voice features such as the extended Geneva Minimalistic Acoustic Parameter Set (eGeMAPS; Eyben et al., 2015) and the Computational Paralinguistics ChallengEs parameter set (ComParE; Schuller et al., 2013).

The features that are included in such sets can be subdivided into three categories: parameters related to frequency aspects (e.g. pitch), parameters related to the energy or amplitude of the signal (e.g. harmonics-to-noise ratio; HNR) and spectral parameters (e.g. harmonic differences). Another common approach to produce a more specialised parameter set is the usage of the unsupervised Bag-of-Audio-Words (BoAW) approach to the best set of features according to a customised codebook quantisation of the low level descriptors (LLDs). In addition, machine learning models have been applied to vocalisations including neural networks in different varieties, testing classification tasks (e.g. adult vs. infant speech, canonical vs. non-canonical utterances; Ebrahimpour et al., 2020; Warlaumont et al., 2010). In our group, we have utilised a machine learning approach (i.e. support vector machines), that focused on automatic preverbal vocalisation-based differentiation between typically developing infants and infants later diagnosed with RTT, FXS or ASD (Pokorny et al., 2016a, 2017, 2022). Studies evaluating acoustic features of early vocalisations or applying machine learning models or neural networks will be referred to as “computational studies” hereafter.

Given recent efforts to perceptually classify preverbal vocal patterns and characterise them acoustically, there is still a lack of synergised information in the field of prodromal or pre-diagnostic development in infants with neurodevelopmental or genetic disorders, especially concerning the pre-babbling phase. Therefore, the current article aimed to (i) outline characteristics of age-specific pre-linguistic vocalisations in the first 5 months of age (i.e. the pre-babbling phase), (ii) summarise computer-based approaches for the automated analysis of physiological and pathological pre-babbling vocalisations, and (iii) compare computer-based approaches on atypical early verbal functions and outline their potential to serve as neurofunctional marker of DDs.

## Methods

To address the above-mentioned issues, we systematically searched the existing literature for (a) characteristics of and (b) state-of-the-art computational and observational methods on prelinguistic vocalisations in infants with DDs. We conducted two rounds of paper extraction and selection, the first one in September 2021 and a second one in February and March 2022 in the following online electronic databases: PubMed, Web of Science, Science Direct, Scopus, and Psy-cINFO using the search strings “infan* AND (prelinguistic OR preverbal OR cooing OR babbling OR vocal) AND (syndrome OR “genetic disorder” OR “developmental disorder”)” and “infan* AND vocal* AND (“computational analysis” OR “acoustic analysis” OR “audio analysis”)”.

Following this initial step, we performed an ancestral search for papers from the retrieved articles and searched Google Scholar for further publications. The retrieved articles were screened by two independent raters (CW and SL). Results were discussed with the co-authors, duplicates were removed, and articles were selected according to the following criteria: (1) peer-reviewed; (2) original studies or reviews and meta-analyses; (3) written in English; and (4) focusing on the pre-babbling age (0 to 5 months) in (4a) typically developing infants and (4b) infants at elevated likelihood for or diagnosed with neurodevelopmental disorders (NDDs), late detected developmental disorders (LDDDs), genetic syndromes, or developmental disorders (DDs). Articles of interest were those based on human coder-based assessments (observational studies) as well as articles on machine learning approaches (computational studies). We intended to focus on spontaneous infant vocalisations and excluded all studies analysing or reporting infant cry or distress vocalisations as well as vocalisations from parent–child interaction paradigms (PCI).

## Results

Our literature selection process led to a total of 27 papers, 17 of which are on pre-babbling in infants diagnosed with neurodevelopmental disorders or genetic syndromes applying observational methods (Table 1). Six articles focused on DS, seven on ASD (one of them also including infants with TSC), three on RTT or the preserved speech variant of RTT (PSV), and one on PWS. Two of the 17 articles reported acoustic features in addition to observational characteristics. The remaining ten articles focused on acoustic features/computational models, three studies applying computational methods on pre-babbling behaviour in TD infants (Table 2) and seven papers discussed the babbling stage in infants later diagnosed with a DD (i.e. ASD, CDS, PSV-RTT, RTT, WS and one study reporting on ASD, FXS, and RTT; Table 2). It is important to note that a differentiation between spontaneous vocalisations vs. vocalisations in interactive settings could not be reliably done for all articles. Thus, against the initially set exclusion criterion, we decided to report all observational studies of this age-range and outlined information on data sampling whenever possible (Tables 1 and 2).

**Fig-1:**
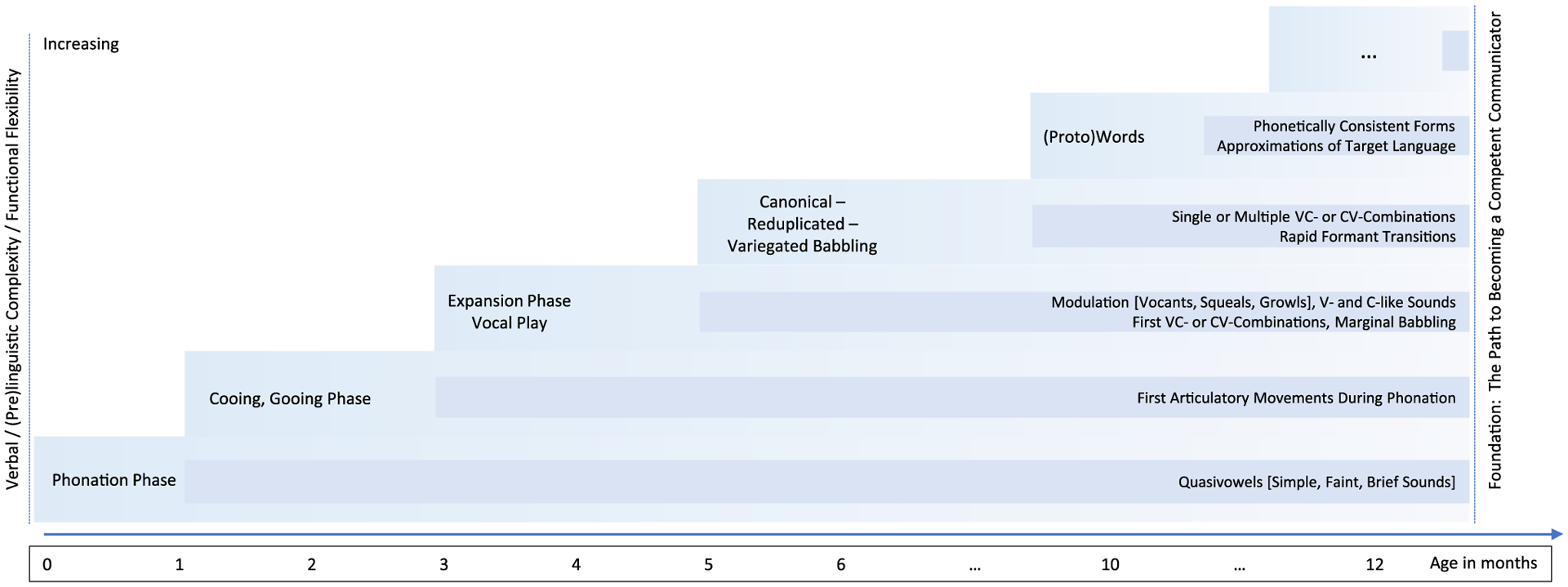
The developing speech-language capacity

Whilst there is a number of studies reporting early physiological development according to the established stage models (Fig. 1), reports of atypical development in infants with neurodevelopmental disorders or genetic syndromes in the younger ages are rare (Table 1). Most of the 17 included studies report on expanded age-bands up to 24 months; very few explicitly investigate the characteristics of early verbal functions emerging in the first 5 months of life (Brisson et al., 2014; Maestro et al., 2002; Pansy et al., 2019; Zappella et al., 2015). Most studies investigate developing verbal functions applying the classical approach of perceptual segmentation-annotation-classification. There is less effort present in delineating acoustic features (such as duration of vocalisations, syllables or phrases, pitch, fundamental frequency (*F*_0_) or intonation contours; Brisson et al., 2014; Lynch et al., 1995a). Observational studies reveal inconclusive results on behavioural differences in pre-babbling vocalisations in infants with DDs and typical development. Compared to TD infants several diverse behaviours have been reported for DD: e.g. longer duration of rhythmic units in infants with DS (Lynch et al., 1995a); divergent intonation contours and less vocal response in interactive settings (Brisson et al., 2014); some participants with ASD failed to achieve the developmental milestone “cooing” (Maestro et al., 2002; Zappella et al., 2015); typical vocalisations interspersed with atypical forceful and/or inspiratory vocalisations in infants with RTT (Marschik et al., 2009); more details on age-specific vocalisations and characteristics of this period are outlined in Table 1.

More advanced methods such as digital measurement instruments and computational analyses open new possibilities for earlier identification of atypical development, as they surpass human capabilities of perception. Most approaches identified aim to describe and investigate trends in the typical development of vocalisations throughout the first 5 months of life. Very early studies focus on a categorical analysis of vocalisations, applying spectral analysis to gain additional insights in addition to the verbal Gestalt-perception (Buder et al., 2008; Lynch et al., 1995a; Oller et al., 2019; Warlaumont et al., 2010). The spectra analysed were acquired through the application of a window function. Most commonly, a fast-Fourier transformation is used to present results as a graphical visualisation, showing the intensity of frequencies at a point in time (Heideman et al., 1985). With the resulting graphical representation, one can visually determine fundamental and formant frequencies (*F*_0_ and *F*_n_ respectively) and the general “shape” of a vocalisation (Bauer & Kent, 1987; Kent & Murray, 1982; Oller et al., 2019). The method of spectrography has been applied in studies over the last 3 decades, finding specific intonation patterns in pre-babbling vocalisations and a developmental trajectory of the *F*_0_ and *F*_n_ (Kent & Murray, 1982). Oller and colleagues used spectrograms to visualise examples of vocants, squeals, growls, and cries at specific ages, providing a visual description of the noise found in the signal as well as other unique features (e.g. *F*_0_ contour) of the analysed classes of utterances (Oller et al., 2019). Another feature, which can be identified through inspection of the spectrogram or the waveform of a vocalisation, is the duration of a single utterance. The duration is used in several studies to gain an understanding of how utterance durations change with age (Apicella et al., 2013; Brisson et al., 2014; Lynch et al., 1995a; Smith & Oller, 1981).

More in depth analyses of audio signals require multi-dimensional parameter sets to provide feature-based representations of the underlying audio segment to a classifier, which can then build an optimal predictor for the classification scheme provided. There are pre-defined parameter sets that are commonly utilised in linguistic and acoustic analyses. Such parameter sets are for example the Computational Pralinguistics ChallengEs parameter set (ComParE; Schuller et al., 2013) or the eGeMAPS (Eyben et al., 2015). These parameter sets consist of low-level descriptors (LLDs). LLDs are parameters that are very closely related to the signal itself (e.g. fundamental frequency *F*_*0*_. loudness). To gain further insights about the general occurrence and statistical behaviour of those LLDs, functionals (e.g. mean, kurtosis, variance) are used on top of these (Schuller & Batliner, 2013).

Yet, in the field of pre-babbling vocalisations, most studies rely on basic features such as duration or fundamental frequency to gain a more in depth understanding of infant vocalisations (Apicella et al., 2013; Brisson et al., 2014; Lynch et al., 1995a; Smith & Oller, 1981). Visual spectrogram analysis has been used to evaluate different vocalisation shapes and help estimate signal to noise ratios in certain vocalisation types (Oller et al., 2019). These approaches, whilst not utilizing advanced computational methods, highlight the importance of particular features for identification of certain vocalisation types and analysis of developmental trajectories. Lynch and colleagues, who focused on a comparison between TD children and children with DS, present the only study that employs a feature-based approach in the analysis of pre-babbling vocalisations in infants with DDs (Lynch et al., 1995a). In this study, the duration of utterances was compared between DS and TD children across respective timelines. For the first 5 months of life, no significant difference was found between TD infants and infants with DS. Nevertheless, the duration of utterances increases until 8 months of age and then decreases until 12 months of age, continuously diverging between TD and DS groups (Lynch et al., 1995a). Although the methodology is not sensitive enough for an accurate differentiation between the two studied groups, it provides a starting point in the identification of possible features that can be used for future analysis of pre-babbling vocalisations (Lynch et al., 1995a). This early phase of verbal development is not yet very well researched in terms of the effectiveness of the aforementioned parameter sets (i.e. ComParE & eGeMAPS). So far, there is a lack of studies applying advanced computational approaches as well as comparative studies that enable rendering a verdict on their applicability (see Table 2). Deep learning approaches have been applied to different settings (e.g. interactive settings, home recordings; Pokorny et al., 2020) of pre-segmented infant audio signals to solve superficial classification tasks (e.g. infant vs. adult, canonical vs. non-canonical). However, none of these studies focused on infants with DDs in the first few months of life (Ebrahimpour et al., 2020; Warlaumont et al., 2010).

Several studies on machine learning approaches applied to vocalisations in the first year of life (pre-babbling and babbling) were identified. In the pre-babbling phase, only three studies utilised approaches beyond the manual analysis of LLDs in the assessment of vocalisations in TD infants (Table 2). To the best of our knowledge, there are no studies available in infants at risk or with a later diagnosis of DDs. These approaches investigate the effectiveness of different neural network architectures (i.e. convolutional neural network, self-organising map and perceptron hybrid network), input features (i.e. spectrograms, waveform, parametric representation), and classification schemes (i.e. infant-directed speech vs. adult-directed speech, infant vs. adult, vocalisation vs. non-vocalisation, canonical vs. non-canonical; vocant vs. squeal vs. growl; Ebrahimpour et al., 2020; Li et al., 2021; Warlaumont et al., 2010). Opposed to that, in the babbling phase, a number of studies analyse verbal capacities utilizing computational approaches (e.g. Pokorny et al., 2018, 2020, 2022). In general, manual analysis of LLDs such as fundamental frequency (F_0_) is not very common for babbling vocalisations. Spectrographic analysis is very often used only for representational purposes, e.g. to represent different syllable types (e.g. Poeppel & Assaneo, 2020). For analysis and detection of atypical development by utilising computational methods, the number of approaches described is limited (Table 2).

## Discussion

Some 40 years ago, the field of early infant vocalisation study was revolutionised with new ways to assess, measure and interpret early development (Koopmans-van Beinum & van der Stelt, 1986; Oller, 1978; Papoušek, 1994; Roug et al., 1989; Stark, 1980). Since then, we have learned a lot about infant prelinguistic development and vocalisation categories. Most studies, however, focused on babbling and the emergence of first words (second half of the first year of life) whilst the pre-babbling phase (first months of life), especially in infants at elevated likelihood for or diagnosed with neurodevelopmental disorders and genetic syndromes, was less researched.

The very early phase of verbal development is mostly described through the achievement of certain milestones (e.g. phonation, cooing, expansion) or via perceptual assignment of infant vocalisations to certain types (e.g. vocant, canonical syllable). Another, albeit still rarely used approach is the description of infant vocalisations through acoustic features (e.g. duration, mean pitch, *F*_0_). Studies have only recently focused on the investigation of quantitative changes of different vocalisation types in the first 5 months of life (Jhang & Oller, 2017; Oller et al., 2013, 2019). However, these studies have not assessed infants with developmental disorders or genetic syndromes so far. Threshold definitions, such as the canonical babbling ratio (CBR) applied in the second half of the first year of life, have to the best of our knowledge, not yet been developed or used for types of pre-babbling vocalisations. For the later stages of development, a number of different approaches to define the onset of certain functions (e.g. canonical babbling) providing similar critical time periods in which milestones are achieved (Lang et al., 2021; Molemans et al., 2012; Oller, 2000), have been proposed. Oller and colleagues (Oller et al., 1998, 1999) reported that delayed onset of canonical babbling is a precursor to later adverse linguistic functioning. Whether precursors of atypical development may already be detected in earlier vocalisations has not yet been investigated. Further research observing typical verbal development is still needed for a basis to understand deviant patterns and trajectories.

Besides pioneering the field of perceptively evaluating infant vocalisations, Oller and colleagues were also at the fore-front to propose semi-automated recording and analytical tools for the assessment of infant vocalisations (e.g. LENA system; Oller et al., 2010). Challenges of recording preverbal data as well as advantages of automated tools for the acquisition and analyses of acoustic features have been increasingly discussed (Pokorny et al., 2020). The aim of this article is not to discuss pros and cons of automated data acquisition approaches but to focus on whether such undertakings have been utilised in the study of infant vocalisations in the first half year of life, in typical cohorts, in individuals at elevated likelihood for DDs, or groups with DDs or pre-/perinatally diagnosed disorders.

When looking beyond behavioural observations and general perceptual evaluations of early infant vocalisations, there is a lack of computational methods that study, substantiate, and support the findings of observational studies. We found that despite the existence of thoroughly tested computational approaches for babbling-vocalisations, there are no attempts to use these methods in the evaluation of pre-babbling vocalisations. These perceptually less salient vocalisations, as compared to canonical babbling, have preferably been studied through simple LLDs such as F_0_ and duration. Only a few studies have used more advanced computational approaches to prove the applicability and value of such approaches in the field of pre-babbling vocalisations (Ebrahimpour et al., 2020; Li et al., 2021; Warlaumont et al., 2010). Besides missing analytical approaches, there is also a lack of standardisation of coding-schemes and datasets, which impedes the comparability of performance between applied computational models in the field of speech-language analysis in the first 5 months of life. Additionally, the sample sizes investigated in observational and computational studies are usually small (i.e. 1–119; see Table 2). Generalisation capabilities of machine learning approaches applied on small dataset sets are questionable. Computational or feature-based approaches are underrepresented in studying pre-babbling vocalisations, especially in infants with NDDs (Brisson et al., 2014; Lynch et al., 1995a). To fingerprint early neurofunctional development and its deviations (Marschik et al., 2017), we need in-depth understanding of physiological functioning as well as disorder specific characteristics. Early verbal development is one domain of interest cluing in the integrity of the developing nervous system. Recent development of analytical tools appear well suited for analysing pre-linguistic vocalisations at pre-babbling age to enhance our insights into emerging early verbal functions. Pioneer work is required to verify computational tools in identifying disorder-specific features in early vocalisations, which may inform future clinical diagnoses and be used for monitoring therapeutic success.

## Figures and Tables

**Table 1:** Studies analysing pre-babbling behaviour in infants with neurodevelopmental disorders or genetic syndromes applying observational methods (ascending order)

Observational studies on pre-babbling in neurodevelopmental disorders or genetic syndromes						
Authors (year of publication)	Condition: sample size (M/F)	Age range (in months)	Data sampling	Behaviours analysed related to pre-babbling vocalisations		Key results
Smith and Oller (1981)	TD: *n* = 9 (7/2) DS: *n* = 10 (5/5)	T1: 0–3 T2: 3–6 T3: 6–9 T4: 9–12 T5: 12–15 T6 (DS): 15–18 T7 (DS): 18–21	Longitudinal Prospective Laboratory audio recordings Every 3 months Interaction sequences 30 min duration	Vocalic productions Consonantal productions Transition from pre-babbling to babbling		Substantial similarities between TD and DS infants in terms of the place of articulation of vowels and consonants Transition to babbling at 6–10 (average: 7.9) months in TD and at 7–12 (average: 8.4) months in infants with DS
Legerstee et al. (1992)	DS: *n* = 8 (4/4)	2–10	Longitudinal Prospective Laboratory video recordings Biweekly (8–24 weeks) Monthly (25–40 weeks) Fixed experimental setup with mother, stranger, and object	Three vocal categories: vocalic (nonspeech-like), melodic (speech-like), emotional (laughing, crying, fussing)		From 17 weeks onwards, vocalisations could be categorised as vocalic, melodic, and emotional; more vocalisations were produced to people than to objects Only from 6 months onwards mother–stranger and active–passive differentiation
Steffens et al. (1992)	TD: *n* = 27 (17/10) DS: *n* = l3 (4/9)	4–18	Longitudinal Prospective Laboratory audio recordings Bimonthly	Four vocalisation types: quasi-resonant syllables, fully resonant syllables, marginal syllables, and canonical syllables		In both TD and DS infants increase of fully resonant and canonical syllables In both TD and DS infants decrease of quasi-resonant and marginal syllables No significant differences between DS and TD infants’ developmental patterns concerning pre-babbling
Lynch et al., (1995b)	TD: *n* = 27 (17/10) DS: *n* = 13 (4/9)	4–18	Longitudinal Prospective Laboratory audio recordings Monthly, merged to 2-month-sampling bins 20 min duration	Four vocalisation types: quasi-resonant syllables, fully resonant syllables, marginal syllables, and canonical syllables Focus on transition to canonical babbling		No details on pre-babbling As a group, infants with DS showed a delayed transition from pre-babbling to babbling and “less stable” canonical babbling
Lynch et al., (1995a)	TD: *n* = 8 (−/−) DS: *n* = 8 (−/−)	T1: 2–4 T2: 6–8 T3: 10–12	Longitudinal Prospective Laboratory audio recordings Monthly 20 min duration		Non-vegetative utterances identified as syllables, utterances, and prelinguistic phrases Perceptual characteristics of syllables: nucleus in isolation (vowel-like sound) or closant (consonant-like sound) and nucleus in sequence	Infants with DS showed similar amounts but longer duration of rhythmic units (syllables, utterances, phrases) when compared to TD infants
Maestro et al. (2001)	TD: *n* = 15 (11/4) ASD: *n* = 15 (10/5)	T1: 0–6 T2: 6–12 T3: 12–18 T4: 18–24	RVA, home recordings One video per child		Vocalisations in interactive settings	Infants with ASD showed deviancies in socio-communicative development in their first month when compared to TD infants No details concerning vocalisation development during the pre-babbling phase
Maestro et al. (2002)	TD: *n* = 15 (9/6) ASD or PDD-NOS: *n* = 15 (10/5)	0–6	RVA, home recordings 10–62 min duration One video per child		Vocalisations, sounds or babbling towards another person Vocalisations, sounds or babbling towards objects	Infants with ASD showed significantly lower rates of vocalising towards another person or object as compared to TD children
Maestro et al. (2005)	TD: *n* = 13 (5/8) ASD: *n* = 15 (11/4)	T1: 0–6 T2: 6–12	RVA, home recordings 10–82 min duration		Vocalisations towards another person (social) Vocalisations towards objects (non-social)	Infants with ASD showed an atypical trajectory of social and non-social activities
Marschik et al. (2009)	PSV-RTT: *n* = 1 (0/1)	0–24	Longitudinal RVA, home recordings Additional: parental diaries, medical history		Vocalisations	Typical vocalisations interspersed with atypical episodes of forceful and/or inspiratory vocalisations
Apicella et al. (2013)	TD: *n* = 9 (−/−) ASD: *n* = 10 (9/1)	T1: 0–6 T2: 6–12	RVA, home recordings Dyadic interaction sequences T1 = 0–6 months: 46 min T2 = 6–12 months: 51 min		Caregiver-infant reciprocity scale (CIRS)^a^: Vocalisations to elicit caregivers’ response Vocalisations to respond to caregiver	Infants with ASD showed similar amounts of vocalisations in the first half year of life compared to TD infants but a significant decrease of total vocalisations from T1 (0–6 months) to T2 (6–12 months)
Marschik et al. (2013)	RTT: *n* = 10 (0/10) PSV-RTT: *n* = 5 (0/5)	0–24	RVA, home recordings Total: 2431 min, typical RTT: 2041 min, PSV-RTT: 390 min		Realisations of age-specific speech-language milestones (cooing, babbling, (proto-) words, word combinations) Exclusion of fixed vocal signals (e.g. crying, laughing), vegetative sounds (e.g. sneezing, coughing)	Infants with RTT or PSV-RTT showed diverging behaviours when compared to TD infants (delayed or non-achievement of milestones)
Brisson et al. (2014)	TD: *n* = 13 (9/4) ASD: *n* = 13 (11/2)	0–6	RVA, home recordings Dyadic interaction sequences		Prosodic analysis: duration, mean pitch, fundamental frequency Analysis of intonation contours Exclusion of non-speech productions (e.g. cries, fusses, laughter)	Infants with ASD produced vocalisations with similar duration and pitch Infants with ASD used simple intonation contours more often; vocalisations contained less complex modulations
Einspieler et al. (2014)	RTT: *n* = 2 (0/2) monozygotic twins	0–22	Longitudinal RVA, home recordings Additionally: parental diaries, medical history Total twin 1: 55 min/38 clips, total twin 2: 54 min/36 clips		Vocalisations	Infants with RTT showed indications of speech-language atypicalities in the first year of life before showing clear signs of regression
Zappella et al. (2015)	Suspected ASD: *n* = 18 (18/0) ASD+ : *n* = 10 ASD−: *n* = 1 TSC: *n* = 7	T1: 1–2 T2: 3–4 T3: 5–6	RVA, home recordings Total: 314 min, 3–11 min per infant per 2-month-range		Unspecific vocalisations (basic sounds or vowel-like sounds) Pleasure vocalisations/laughter Cooing (melodic modulated vocalisations)	The majority of infants later diagnosed with ASD or TSC showed age-adequate vocalisations (i.e. cooing and pleasure vocalisations) during the first 6 months Six infants out of the whole sample did not develop cooing during the first 6 months
Chericoni et al. (2016)	TD: *n* = 10 (8/2) ASD: *n* = 10 (9/1)	T1: 0–6 T2: 6–12 T3: 12–18	Longitudinal RVA, home recordings Dyadic interaction sequences Each participant 3 min per time period T1, T2, T3 Total: 142 sequences (T1: 47, T2: 47, T3: 48 sequences)		Vocalisations consisting of vowels/non-reduplicated consonants and vowels Transition to babbling and words Exclusion of non-speech sounds (such as squeals, yells, growls, or grunts)	At T1 (0–6 months) no differences in the rate of vocalisations per minute were observed between infants with ASD and TD infants
Pansy et al. (2019)	PWS: *n* = 1 (1/0)	0–6	Longitudinal Prospective Audio-video recording at 27 weeks 10 min duration		Classification of vocalisations according to the Stark Assessment of Early Vocal Development-Revised (SAEVD-R)^b^	Age-adequate distribution of SAEVD-R-levels with high proportion of level-3 vocalisations Vocalisation quality was observed to be monotonous and with peculiar harmonic structure Transition to level-4 vocalisations (canonical babbling) was not reached during the first 6 months
Onnivello et al. (2021)	DS: *n* = 14 (38/36)	4–18	Cross-sectional Prospective Laboratory test situation (approximately 40 min)		BSID-III^c^ Expressive communication scale: Vocalisations of mood Two vowel sounds Two consonant sounds Further milestones (babbling and word use)	Most prominent delays in development of infants with DS were observed in the communicative capabilities The first 6 months of vocal development were similar in infants with DS and TD infants concerning vocalisation of mood and social vocalising The onset of more speech-like communicative functions (i.e. babbling and first words) was delayed in infants with DS

*ASD* autism spectrum disorder, *DS* Down syndrome, *PDD-NOS* Pervasive Developmental Disorder-Not Otherwise Specified, *PSV-RTT* preserved speech variant–Rett syndrome, *PWS* Prader-Willi syndrome, *RTT* Rett syndrome, *RVA* retrospective video analysis, *TD* typically developing, *TSC* tuberous sclerosis complex

a Only verbal scale attributes of CIRS (for details see Apicella et al., 2013)

b SAEVD-R (for details see Nathani et al., 2006)

c Bayley Scales of Infant and Toddler Development Third Edition (Bayley, 2006)

**Table 2:** Studies applying computational methods on pre-babbling behaviour in typically developing infants and on babbling behaviour in infants with neurodevelopmental disorders or genetic syndromes (ascending order)

Computational studies on pre-babbling in TD					
Authors (year of publication)	Condition: sample size (M/F)	Age range (in months)	Data sampling	Behaviours analysed related to pre-babbling vocalisations	Methods/results
Warlaumont et al. (2010)	TD: *n* = 6 (2/4)	T1: 3–6 T2: 6–9 T3: 9–12	Longitudinal Prospective Laboratory audio–video recordings Interaction and non-interaction sequences At least two 20-min sessions per age interval, first 49 utterances coded	Interactive and spontaneously produced vocalisations Vocalisation types: vocants, squeals, and growls Exclusion of crying, distress, vegetative sounds	Segmented infant non-cry vocalisations with a duration of 1 s were transformed into a spectrogram using the fast Fourier transformation and passed into the self-organising map with a 4 × 4 grid which was then used as the input for a single-layer perceptron Classification of vocalisation types: vocants, squeals, and growls with an accuracy of 0.55
Ebrahimpour et al. (2020)	TD: *n* = 15 (−/−)	3–18	Longitudinal Prospective LENA^a^ all day recordings Three 5-min periods of active vocalisations	Vocalisation categories: laugh/cry, non-canonical babbling (vowels or consonants), canonical babbling (well-structured syllables with consonants and vowels)	The dataset presented by Warlaumont et al. (2010) was used Two end-to-end convolutional neural networks using time-domain waveform data as input were used. Different conditions were tested: infant vs. adult, vocalisation vs. non-vocalisation, infant-directed speech (IDS) vs. adult-directed speech (ADS), laugh/cry vs. canonical/non-canonical vs. IDS vs. ADS, laugh/cry vs. canonical vs. non-canonical vs. IDS/ADS, laugh/cry vs. canonical vs. non-canonical vs. IDS vs. ADS Classification accuracy of 0.41–0.94 in the above-mentioned cases was obtained (above chance) Non-canonical babbling vs. canonical babbling and IDS vs. ADS changed as a function of age and became more accurate with increasing age
Li et al. (2021)	TD: *n* = 119 (−/−)	3–12	Longitudinal Prospective Home and laboratory audio–video recordings Home recordings: LENA^a^ Lab recordings: semi-structured interaction sequences and still face procedure	Vocalisation types: cry, fuss, laughter, babbling, screech	1582 spectral and prosodic features were used Multiple different machine learning methods (i.e. linear discriminant analysis, convolutional neural network with self-attention, feed-forward fully-connected neural network) were applied to differentiate infant and adult vocalisation types From three types of classifiers evaluated, convolutional neural network with self-attention achieved the highest classification accuracy of 87%
Computational studies on babbling in neurodevelopmental disorders or genetic syndromes					
Sohner and Mitchell (1991)	CDS: *n* = 1 (−/−)	8–26	Longitudinal Prospective Audio recordings	Comfort state vocalisations (fundamental frequency, intonation contours)	Analysis of the fundamental frequency of non-cry vocalisation across the period of 8–26 months were performed High fundamental frequency in comfort state vocalisations (521 – 622 Hz) with no downward trend as can be observed in TD infants with a fundamental frequency of 410 Hz at 8 months decreasing to 310 Hz at 26 months Limited inter-utterance variation of fundamental frequency with a predominance of falling intonation contours
Pokorny et al. (2016a)	TD: *n* = 4 (0/4) RTT: *n* = 4 (0/4)	6–12	RVA, home video recordings Total:> 1000 min RTT: 2199 vocalisation TD: 2479 vocalisation	Vocalisations (vocal breathing groups) Exclusion of vegetative sounds (e.g. hiccups, sneezes)	Feature representation of the signal using the ComParE^c^ Classification of TD vs. RTT using a support vector machine Comparison of two approaches: audio signal with speaker normalisation and without speaker normalisation (speaker normalisation being infant dependent normalisation to the interval [0,1]) Classification accuracy was 76.5% for classification of TD infants vs. infants with RTT vocalisations using speaker normalisation
Pokorny et al. (2016b)	ASD: *n* = 14 (13/−) IT FXS: *n* = 1 (1/−) AT RTT: *n* = 2 (−/2) AT RTT: *n* = 2 (−/2) DE RTT: *n* = 5 (−/5)UK TD: *n* = 9 (5/4) AT	7–12	RVA, home video recordings Total:> 1000 min ASD: 696 vocalisations FXS: 87 vocalisations RTT: 2585 vocalisation TD: 1535 vocalisations	Voice-active segments Vocalisations/utterances (vocal breathing groups) Exclusion of vegetative sounds (e.g. hiccups, sneezes)	Multiple different automatic voice detection algorithms (i.e. cVaDV1, hidden Markov models, support vector machines, and random forests) were compared against manual annotation The best performance was obtained using the hidden Markov model with the Gaussian mixture model achieving acoustic event detection accuracy of 0.29
Pokorny et al. (2017)	TD: *n* = 10 (5/5) ASD: *n* = 10 (5/5)	10	Longitudinal Prospective Laboratory audio–video recordings Parent-child interaction sequences 12 min duration	Vocalisations Types of vocalisations more complex than single canonical syllables, based on Stark Assessment of Early vocal development-Revised (SAEVD-R)^b^ Exclusion of vegetative sounds	eGeMAPS^d^ features were compared between infants with ASD and TD infants applying Mann–Whitney *U* test A linear kernel support vector machine and a single-layer bidirectional long-short term memory neural network were tested For 54 out of 88 extracted features a significant difference between vocalisations of TD infants vs. infants with ASD was found Both approaches (i.e. linear kernel support vector machine and single-layer bidirectional long short-term memory neural network) achieved an accuracy of 75% in a subject-wise identification of ASD and TD infants
Pokorny et al. (2018)	PSV-RTT: *n* = 1 (−/1)	7–12	RVA, home video recordings Total: 363 vocalisation (61 min)	Typical vs. atypical vocalisations Exclusion of vegetative sounds	ComParE^c^ features were compared with the atypicality factor calculated in the listener experiment (proportion of listeners that have rated the vocalisation as atypical) Atypicality was mainly related to the characteristic“timbre” and prosodic, spectral, and voice quality features in the acoustic domain More than 50% of the vocalisation of the infant with PSV-RTT were rated as atypical by at least one listener
Pokorny et al. (2020)	TD: *n* = 3 (−/−) RTT: *n* = 3 (−/−)	6–12	RVA, home video recordings Total: 3502 vocalisation	Typical vs. atypical vocalisations	Two parameter sets, eGeMAPS^d^ and ComParE^c^, and a linear kernel support vector machine were used to classify typical vs. atypical vocalisations The best approach reached a performance of 0.879 unweighted average recall using the eGeMAPS^d^ feature set
Ouss et al. (2020)	TD: *n* = 19 (−/−) WS: *n* = 32 (−/−) Outcome 48 M: WS with ASD/ID: *n* = 10 WS without ASD/ID: *n* = 22	9–12	Prospective Laboratory audio–video recordings 3 sequences of 3-min interaction sequences (free play without toy, free play with toy, singing to baby)	Infant vocalisations (babbling vocalisation, laugh, and cry) Atypical vocalisations (other noise)	Annotation of mother-infant speech turn taking Reduction of dataset dimensionality using principal component analysis Classification of TD vs. WS and WS with ASD/ID vs. WS without ASD/ID using a decision tree with a depth of one The approach achieved an accuracy of 76% when classifying between infants with WS and TD infants and an accuracy of 81% when distinguishing between WS with ASD and WS without ASD based on assessed vocalisation types It was found that especially in the classification of WS with ASD and WS without ASD, synchronicity and reciprocity were important factors in the interactive setting

*ASD* autism spectrum disorder, *AT* Austria, *CDS* Cri-du-Chat syndrome, *DE* Germany, *FXS* fragile X syndrome, *ID* intellectual disorder/intellectual disability, *IT* Italy, *PSV-RTT* preserved speech variant–Rett syndrome, *RTT* Rett syndrome, *RVA* retrospective video analysis, *TD* typical development/typically developing, *UAR* unweighted average recall, *UK* United Kingdom, *WS* West syndrome

a LENA (for details see Oller et al., 2010)

b SAEVD-R (for details see Nathani et al., 2006)

c ComParE (for details see Schuller et al., 2013)

d eGeMAPS (for details see Eyben et al., 2015)
